# Deep learning‐based pathology image analysis predicts cancer progression risk in patients with oral leukoplakia

**DOI:** 10.1002/cam4.5478

**Published:** 2023-01-31

**Authors:** Xinyi Zhang, Frederico O. Gleber‐Netto, Shidan Wang, Roberta Rayra Martins‐Chaves, Ricardo Santiago Gomez, Nadarajah Vigneswaran, Arunangshu Sarkar, William N. William, Vassiliki Papadimitrakopoulou, Michelle Williams, Diana Bell, Doreen Palsgrove, Justin Bishop, John V. Heymach, Ann M. Gillenwater, Jeffrey N. Myers, Renata Ferrarotto, Scott M. Lippman, Curtis Rg Pickering, Guanghua Xiao

**Affiliations:** ^1^ Quantitative Biomedical Research Center, Department of Population and Data Sciences University of Texas Southwestern Medical Center Dallas Texas USA; ^2^ Department of Head & Neck Surgery The University of Texas MD Anderson Cancer Center Houston Texas USA; ^3^ Faculdade Ciências Médicas de Minas Gerais (FCM‐MG) Universidade Federal de Minas Gerais Belo Horizonte Brazil; ^4^ Department of Oral Surgery and Pathology, School of Dentistry Universidade Federal de Minas Gerais Belo Horizonte Brazil; ^5^ Department of Diagnostic and Biomedical Sciences The University of Texas Health Science Center at Houston School of Dentistry Houston Texas USA; ^6^ Department of Thoracic‐Head & Neck Medical Oncology The University of Texas MD Anderson Cancer Center Houston Texas USA; ^7^ Hospital BP A Beneficência Portuguesa de São Paulo Sao Paolo Brazil; ^8^ Global Product Development Oncology, Pfizer, Inc. New York New York USA; ^9^ Department of Anatomical Pathology The University of Texas MD Anderson Cancer Center Houston Texas USA; ^10^ Department of Pathology City of Hope Duarte California USA; ^11^ Department of Pathology University of Texas Southwestern Medical Center Dallas Texas USA; ^12^ Department of Medicine University of California San Diego San Diego California USA; ^13^ Department of Bioinformatics University of Texas Southwestern Medical Center Dallas Texas USA

**Keywords:** carcinogenesis, convolutional neural network, disease progression, oral leukoplakia, patient prognosis, precancer, whole slide imaging

## Abstract

**Background:**

Oral leukoplakia (OL) is associated with an increased risk for oral cancer (OC) development. Prediction of OL cancer progression may contribute to decreased OC morbidity and mortality by favoring early intervention. Current OL progression risk assessment approaches face large interobserver variability and is weakly prognostic. We hypothesized that convolutional neural networks (CNN)‐based histology image analyses could accelerate the discovery of better OC progression risk models.

**Methods:**

Our CNN‐based oral mucosa risk stratification model (OMRS) was trained to classify a set of nondysplastic oral mucosa (OM) and a set of OC H&E slides. As a result, the OMRS model could identify abnormal morphological features of the oral epithelium. By applying this model to OL slides, we hypothesized that the extent of OC‐like features identified in the OL epithelium would correlate with its progression risk. The OMRS model scored and categorized the OL cohort (*n* = 62) into high‐ and low‐risk groups.

**Results:**

OL patients classified as high‐risk (*n* = 31) were 3.98 (95% CI 1.36–11.7) times more likely to develop OC than low‐risk ones (*n* = 31). Time‐to‐progression significantly differed between high‐ and low‐risk groups (*p* = 0.003). The 5‐year OC development probability was 21.3% for low‐risk and 52.5% for high‐risk patients. The predictive power of the OMRS model was sustained even after adjustment for age, OL site, and OL dysplasia grading (HR = 4.52, 1.5–13.7).

**Conclusion:**

The ORMS model successfully identified OL patients with a high risk of OC development and can potentially benefit OC early diagnosis and prevention policies.

## Graphical abstract

We developed a deep learning image analysis model using a convolutional neural network to predict the risk of oral cancer development from whole slide images in a patient with oral leukoplakia. The model successfully discriminated patients into two groups with distinct risk for oral cancer development, which represents a potential ancillary tool for the management of oral leukoplakia and may contribute to the early detection and treatment of oral cancer.

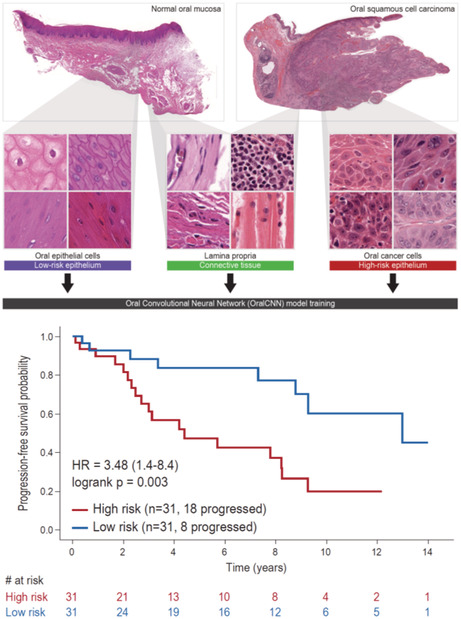

## 1 INTRODUCTION

The concept of premalignancy was introduced more than two centuries ago by a European panel of physicians suggesting that some histologic changes may take place before the onset of cancer.^1^ In the oral cavity, precursor lesions are named oral potentially malignant disorders (OPMD), characterized by visual changes to the oral mucosa, usually white or red patches, which are associated with increased risk for oral cancer (OC) development. Oral leukoplakia (OL) is the most common type of OPMD, for which the malignant transformation rate ranges from 1% to 3% per year.^2^, ^3^

An estimated half million new cases of OC are diagnosed globally each year, with more than 300,000 deaths.^4^ Despite oncological treatment advances, the prognosis for OC patients remains poor, especially for patients diagnosed at advanced disease stages. Early OC detection is critical for therapeutic success and satisfactory quality of life.^5^ Yet, most OC patients are diagnosed at an advanced stage, at least partially due to our limited ability to determine which and when OPMD are at higher risk for malignant progression.^6^, ^7^

Currently, OL malignant transformation risk is estimated by histopathological evaluation of epithelial dysplasia, which involves the detection of architectural alterations and cytological atypia and their extension across the oral epithelial tissue.^8^, ^9^ The World Health Organization proposes a three‐tier OL grading scheme (mild, moderate, and severe) for dysplastic lesions, but due to low accuracy and low reproducibility, simplified binary systems have also been proposed.^10^, ^11^, ^12^ Importantly, the evaluation of epithelial dysplasia is inevitably subjective, resulting in great inter‐ and intraexaminer variability in the interpretation of the presence, degree, and significance of the criteria.^8^, ^13^ Most OL diagnosed with dysplasia never progress to OC in the life of the patient and the time‐to‐progression (TTP) in those that do is not predictive of subsequent invasive disease. Furthermore, various reactive and regenerative changes in the oral epithelium, secondary to trauma and chronic inflammatory ulcerations, closely mimic mild to moderate dysplasia. Hence, a more objective and efficient grading system is needed such that risk stratification of OL patients is suitable for guiding disease management decisions.

Since the introduction of whole slide scanners in 1990, technology has evolved, allowing the creation of digital histology images that retain high levels of detail.^14^ In parallel, the development of deep learning—a newer branch of artificial intelligence (AI)—technologies has provided opportunities to design automated learning algorithms to examine these high‐definition histologic images and potentially benefit the practice of surgical pathology.^15^, ^16^, ^17^, ^18^, ^19^ Some deep learning algorithms have achieved performance comparable to pathologists in tasks such as the detection and segmentation of tumor regions^20^, ^21^ and the identification of metastatic foci in lymph nodes.^22^, ^23^, ^24^ Among deep learning techniques, convolutional neural networks (CNNs) have been the most successful and have been widely used for a variety of applications including image segmentation and classifications.^16^, ^25^ Through its deep network structure, CNNs can learn from the data sets to extract highly predictive image features and make accurate predictions independent of clinical intervention.^26^, ^27^

In this way, we believe that CNN can be an effective tool for the identification of morphological features associated with malignant progression risk in OL. We hypothesized that OL with a higher risk of cancer progression might exhibit morphological features that resemble OC tissue and that a CNN‐based model would be able to identify such features by analyzing and comparing OC and nondysplastic oral mucosa images. To test this hypothesis, we developed OMRS (oral mucosa risk stratification), a CNN‐based deep learning model that uses images of Hematoxylin and Eosin (H&E) stained tissue as input. This model was initially trained with images of nondysplastic oral mucosa and oral squamous cell carcinoma to create a cancer progression risk score based on the identification of morphological differences between epithelial cells in these two types of tissues. The model was then applied to OL histopathological slides, assuming that OL epithelium with morphological similarity to OC has a higher risk of cancer progression. We demonstrated that OMRS can predict time‐to‐progression more effectively than the three‐tier World Health Organization (WHO) OL classification system, and it performs on par with newer binary systems in terms of disease progression risk assessment. We expect that prospective improvements to OMRS, by the addition of new layers of morphological complexity into the model, could improve the accuracy of OL malignant progression risk assessment and potentially be used to tailor surveillance intervals and treatment decisions for leukoplakia patients.

## 2 METHODS

### 2.1 Training datasets

The OMRS model was developed on hematoxylin and eosin (H&E)‐stained sections of 38 oral mucosal biopsies. Among these tissues, 13 tissue sections consist of nondysplastic oral epithelium, which was retrieved from the archives of the Oral Pathology Service of the School of Dentistry at Universidade Federal de Minas Gerais, Brazil (Table S1). This cohort included biopsies of the clinically normal oral epithelium of oral mucosal diseases with pathologic changes limited to the lamina propria while preserving the normal architecture and cytology of the oral epithelium. The remaining 25 samples were H&E‐stained images of oral squamous cell carcinomas (OSCC) randomly selected from The Cancer Genome Atlas (TCGA) Head and Neck Squamous Cell Carcinoma (HNSCC) tissue imaging data set^28^ (https://wiki.cancerimagingarchive.net/display/Public/TCGA‐HNSC, accessed early 2019. Table S2), with inclusion criteria of oral cavity location and human papillomavirus (HPV) negative. All H&E‐stained slides for model development were scanned or available at 40× magnification.

Each slide/image was evaluated by an expert oral pathologist (F.O.G‐N) in order to annotate areas of technical artifacts that should be excluded and to annotate the regions of the epithelium (tumor or nondysplastic), connective tissue, and background.

### 2.2 OMRS model development

To develop the model using nondysplastic oral epithelium and OSCC tissue slides, image patches measuring 300 × 300 pixels (40×) of four classes (nondysplastic epithelium, cancerous epithelium, connective tissue, and background) were extracted from the annotated regions and randomly divided into training, validation, and testing sets. Image patches from the same slide were always assigned to the same set. The model development dataset size is shown in Table 1.

**TABLE 1:** The number of image patches used to develop the OMRS model. Showing the number of 300 × 300 pixel patches of each class in training/validation/testing sets.

Model development phase	Tissue type	Epithelium	Connective tissue	White background
Training	OSCC	2419	1207	992
	Oral mucosa	2981	1491	649
Validation	OSCC	451	337	343
	Oral mucosa	709	444	138
Testing	OSCC	513	296	288
	Oral mucosa	739	247	181

**FIGURE 1:**
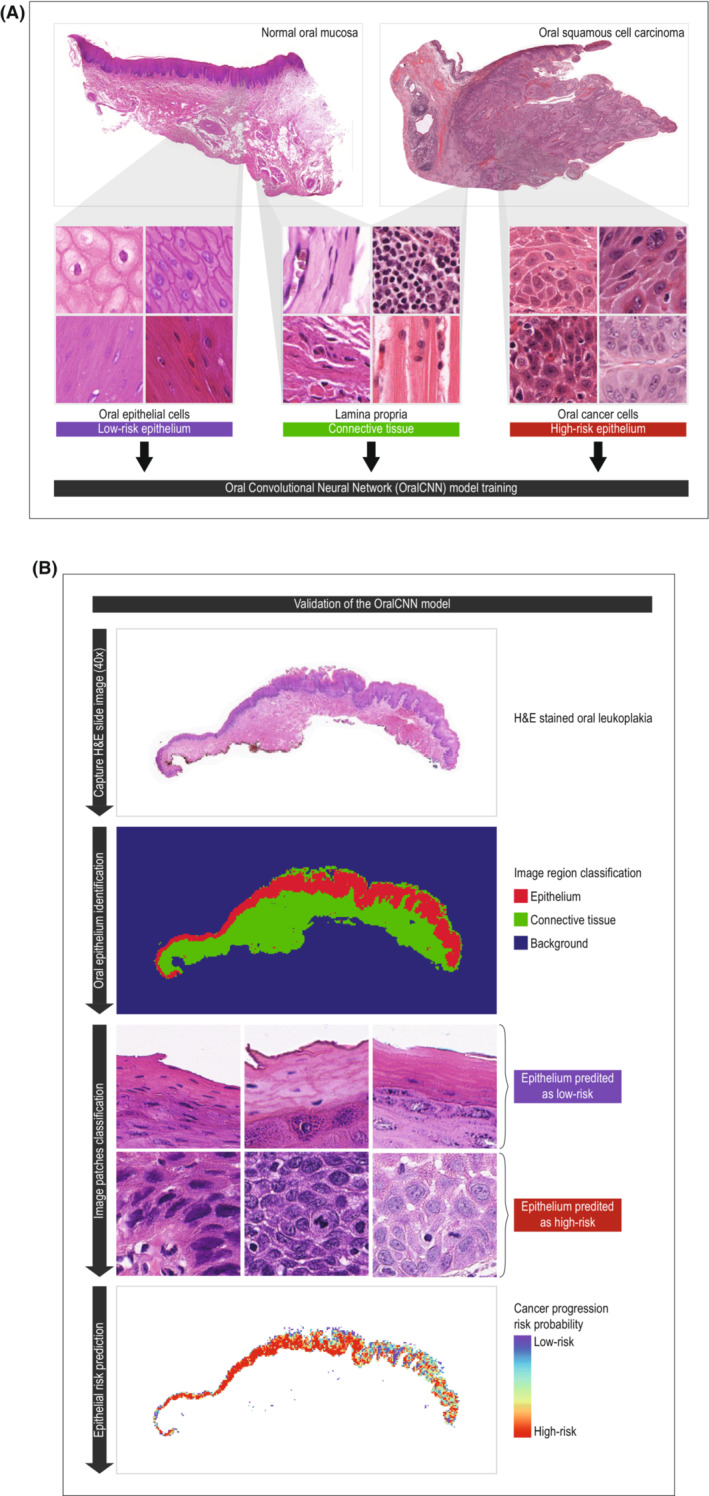
(A) Flow chart of the OMRS model training process. Image patches of high‐risk epithelium, low‐risk epithelium, connective tissue, and white background were extracted from nondysplastic epithelium or oral cancer slides to establish the model. (B) The model predicted on all the slides and generated the prediction heatmap, including the tissue classification heatmap and cancer progression risk probability heatmap.

The approach used for OMRS model development is summarized in Figure 1A. The OMRS model adapted a modified Inception (V3) architecture,^29^ a type of CNN‐based deep learning model. The model was fine‐tuned using our training data set including nondysplastic oral epithelium and OSCC tissue, as described above. The model took as input an image patch and output a patch‐level probability of the four classes. Detailed methodology is described in Appendix S1.

To test the model on the image patch level, we used the test dataset with size as described in Table 1. Probabilities of being in each of the four classes were predicted by the OMRS model and recorded. Slide‐level prediction heatmaps were also generated for each nondysplastic and OSCC tissue pathology slide, where each pixel represented the class of the image patch with the highest probability at that location.

### 2.3 Application of the OMRS model on leukoplakia slides

To evaluate the prognostic performance of the OMRS model on OL patients, we retrieved H&E slides of OL cases biopsied and followed up at the Department of Head and Neck Surgery at The University of Texas MD Anderson Cancer Center. This cohort included 62 patients with clinical OL diagnoses without evidence of concurrent cancer. H&E‐stained slides were reviewed by pathologists and scanned at 40×. In order to assess the prognostic performance of the OMRS model in identifying patients with a high risk of progression to OC, we retrieved demographical and clinical data, including time‐to‐progression (TTP) to oral cancer, from patient clinical charts. OL histopathological grading was performed by an oral pathologist (N.V.) according to the WHO Classification of Tumors.^30^

The OL H&E‐stained tissue images were scanned at 40× magnification. A 300 × 300 pixel window was slid over the entire scanned slide to extract image patches for the OMRS model without overlapping between any adjacent windows. For each image patch, probabilities of being in different classes (nondysplastic and cancerous epithelium, deemed as one class—epithelium, also a second class was defined and included stroma and white background) were predicted and recorded. Based on the prediction, a heatmap was generated for each pathology slide, where each pixel represented the class of the image patch with the highest probability at that location (Figure 1B). From the heatmap, areas predicted as epithelium were used in further analysis. OL epithelium image patches were then evaluated to predict the probability of an OL epithelium patch being either “nondysplastic‐like” or “tumor‐like” by the OMRS‐trained model, which quantified the resemblance of the OL epithelium to tumor or nondysplastic oral mucosa epithelium. We used this predicted probability as the tumor progression risk score for each tissue patch.

### 2.4 Statistical analysis

To determine the progression risk for each slide, we used the median model‐predicted probability value of all the epithelium patches on the slide as the slide‐level risk score. Patients were categorized into two equal‐sized groups—low‐ and high‐risk groups—according to their individual slide‐level risk score in relation to the median risk score of the cohort.

Kaplan–Meier plots were used to summarize the progression‐free survival curves of the patients in predicted low‐ or high‐risk groups. Univariate Cox regression models were used to evaluate the association between clinicopathological variables and TTP. A multivariate Cox proportional hazard (CoxPH) model was used to evaluate the association between the predicted risk score and patient TTP‐adjusted relevant variables. The results were considered significant if the resulting two‐tailed *p* value was less than 0.05.

### 2.5 Ethics

Written informed consent was obtained from patients included in the study. This study was approved by the Institutional Review Board (IRB).

## 3 RESULTS

### 3.1 CNN model distinguishes tissue type

**FIGURE 2:**
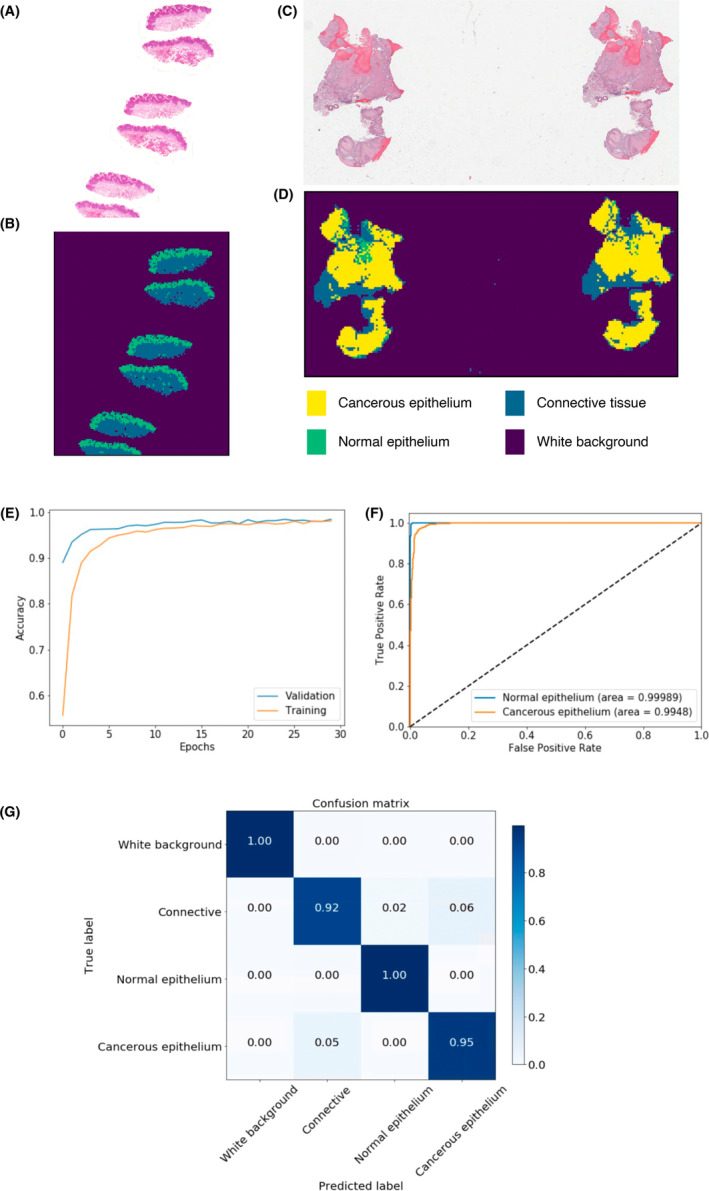
OMRS model training results. (A–D) Example results of image‐level region detection results on nondysplastic and tumor epithelium pathology images. Here (A) and (C) are the original images of nondysplastic and tumor slides, and (B) and (D) are predicted region labels. Yellow: tumor epithelium region; green: nondysplastic epithelium region; blue: connective tissue region; purple: white background region. Each point in the region label maps and cancerous epithelium probability heatmaps corresponds to a 300 × 300‐pixel image patch in the original 40× image. (E) OMRS model learning curves in both training and validation image patch data sets. Epoch is one learning pass of all the training patches. (F) Receiver‐operating characteristic (ROC) curve of nondysplastic epithelium and tumor epithelium classes. (G) Confusion matrix of the trained OMRS model for image patch classification in test data set.

After the training process (Figure 2E–G), the OMRS model showed an overall prediction accuracy in the testing set of 95.4%; the accuracy was 94.7% for tumor epithelium patches and 98.0% for nondysplastic epithelium patches. Receiver‐operating characteristic (ROC) curve showed the area under the curve (AUC) was 0.992 for the tumor and 0.999 for nondysplastic epithelium. Example results of slide‐level region detection results are shown in Figure 2A–D. These results showed that the model was successfully trained with the ability to distinguish nondysplastic epithelium from tumor epithelium very accurately.

### 3.2 Risk score predicts cancer‐free survival

The clinical characteristics of OL patients included in the study are described in Table 2. Patients were followed up for a mean time of 5 ± 4.14 years (minimum of 0.12 and maximum of 14.0 years) after performing the diagnostic biopsy used in the study.

**TABLE 2:** Clinical characteristics of the prognostic evaluation cohorts.

Variables	*n* (%)	No progression	OSCC progression	*p* value
		*n* = 36	*n* = 26	
Gender				
Female	30 (48.4)	14 (22.6)	16 (25.8)	0.123^a^
Male	32 (51.6)	22 (35.5)	10 (16.1)	
Age				
Mean ± SD	55.9 ± 12.0	59.1 ± 9.36	51.5 ± 14.0	0.038^c^
Prior oral cancer				
No	44 (70.1)	27 (43.5)	17 (27.4)	0.571
Yes	18 (29.0)	9 (14.5)	9 (14.5)	
OL dysplasia				
No dysplasia	5 (8.1)	3 (5.26)	2 (3.5)	0.185^b^
Mild	20 (32.3)	16 (28.1)	4 (7.0)	
Moderate	21 (33.9)	11 (19.3)	10 (17.5)	
Severe	11 (19.3)	5 (8.8)	6 (10.5)	
AVEH				
Present	16 (28.1)	12 (21.0)	4 (7.0)	0.236^a^
Absent	41 (71.9)	23 (40.4)	18 (31.6)	
OL site				
Tongue	43 (69.4)	20 (32.3)	23 (37.1)	0.079^b^
Gingiva	6 (9.7)	4 (6.4)	2 (3.23)	
Buccal mucosa	5 (8.1)	5 (8.1)	0	
Floor of the mouth	3 (4.8)	2 (3.2)	1 (1.6)	
Palate	3 (4.8)	3 (4.8)	0	
Oropharynx	2 (3.2)	2 (3.2)	0	
OMRS model risk classification				
Low risk	31 (50.0)	23 (37.1)	8 (12.9)	0.019^a^
High risk	31 (50.0)	13 (21.0)	18 (29.0)	

Abbreviation: AVEH, atypical verrucous epithelial hyperkeratosis.

^a^ Fisher's exact test.

^b^ Pearson Chi‐Square.

^c^ Wilcoxon 2‐Sample Test.

Twenty‐four cases of OL (41.9%) progressed to OC, within 4.4 ± 3.5 years of the initial biopsy (minimum of 0.12 and maximum of 13.0 years). Associations between OC progression and clinicopathological variables are described in Table 2. Malignant progression was significantly associated with younger age (progressors age 51.5 ± 14.0 vs. nonprogressors age 59.1 ± 9.36, Wilcoxon *p* value = 0.038), and with OL located in the tongue (23 out of 43 developed OC, 53.5% progression) compared with the other combined sites (3 out of 19 developed OC, 15.8% progression, Fisher's exact test *p* = 0.006). OL dysplasia grading was not associated with OC progression.

The OMRS risk classification was significantly associated with OC progression. Among OL patients categorized as high risk, 58.1% (18 out of 31) developed OC, whereas only 25.8% (8 out of 31) of the low‐risk patients developed OC (Fisher's Exact test, *p* = 0.019). OL patients classified as high‐risk were 3.98 (CI 95% 1.36–11.7) times more likely to develop OC than low‐risk ones.

**FIGURE 3:**
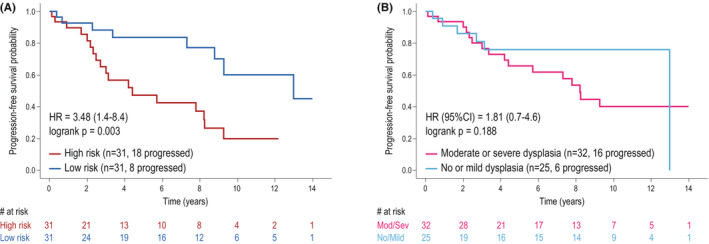
(A) Oral cancer progression‐free survival according to the OMRS model‐predicted risk values. Kaplan–Meier plot shows a significant decrease in progression‐free survival for oral leukoplakia patients classified as “high risk” compared with leukoplakia patients classified as “low risk” (high‐ vs. low‐risk HR = 3.48, 95% CI = 1.44–8.40, *p* value = 0.003). (B) Oral cancer progression‐free survival according to OL dysplasia grading was not significantly associated with survival (HR = 1.81, 95% CI = 0.70–4.65, *p* value = 0.188).

TTP was significantly different between high‐ and low‐risk groups (*p* = 0.003). Low‐risk OL patients had a significantly longer TTP compared with those of the high‐risk group (Figure 3). The 5 and 10 years OC progression probabilities for high‐risk patients were 52.5% (36.1–68.3) and 71.8% (52.4–85.4), respectively, whereas, for the low‐risk group, the probabilities were 21.2% (10.6–38.4) and 34.9% (18.4–55.9). Interestingly, TTP was not different among samples grouped according to their dysplasia grade (no and mild dysplasia versus moderate and severe dysplasia) (Figure 3B).

Univariate analyses showed that age (HR = 0.97, 0.94–1.0 CI 95%), OL site (HR = 4.1, 1.22–13.8 CI 95%), and OMRS risk classification (HR = 3.48, 1.44–8.40 CI 95%) were the only factors significantly associated with TTP. OL dysplasia grading was not associated with TTP (HR = 1.81, 0.7–4.65 CI 95%), but it was also included in the multivariate model since it is considered an important variable for OC progression risk (Table 3). The multivariate analysis showed that the OMRS risk score was the only factor significantly associated with OC progression in OL after adjustment for other variables (HR = 4.52, 1.49–13.7 CI 95%) (Table 3).

**TABLE 3:** Multivariate analysis to adjust OMRS model with clinical variables using Cox proportional hazard model in OL cohort (*N* = 62).

			Univariate analysis			Multivariate analysis		
Variables			HR	CI 95% (low‐high)	Wald test *p* value	HR	CI 95% (low‐high)	Wald test *p* value
OMRS model risk	High risk	Low risk	3.48	1.44–8.40	0.005	4.52	1.49–13.7	0.008
Age			0.97	0.94–1.00	0.042	0.17	0.02–1.85	0.133
Gender	Male	Female	0.83	0.38–1.85	0.660	‐	‐	‐
Prior OSCC	Yes	No	1.29	0.57–2.91	0.547	‐	‐	‐
AVEH	Present	Absent	0.64	0.22–1.90	0.426	‐	‐	‐
OL site	Tongue	Another site	4.10	1.22–13.8	0.022	4.83	0.98–23.8	0.053
OL dysplasia	No/Mild dysplasia	Moderate/Severe dysplasia	1.81	0.70–4.65	0.219	0.85	0.29–2.47	0.770

## 4 DISCUSSION

In this study, we presented the OMRS model, designed to predict OC development risk using H&E‐stained OL slides. The model demonstrated an encouragingly powerful ability to discriminate OL with higher potential for malignant progression from those cases with lower cancer progression risk, serving as an independent and objective prognostic tool different from current practices. To the best of our knowledge, this is the first deep learning study regarding OL pathology H&E image analysis.

Implementation of traditional epithelial dysplasia grading systems used to estimate malignant progression risk of OL requires a dedicated/specially trained expert pathologist, and still suffers from high inter‐ and intraobserver variability with poor reproducibility. Modification of the traditional three‐tier OL grading system to a binary classification has improved the grading system's accuracy. Within the three‐tier grading system, the malignant transformation rate (MTR) for OL classified as mild and moderate has been reported as ranging from 5% to 12%, whereas among severe cases, the MTR is around 25%.^31^, ^32^ On the other hand, within binary systems, OL classified as high risk and low risk have an MTR of 58% and 13% respectively.^10^ In our OMRS model, high‐risk patients have an MTR of 58.1%, indicating that it outperforms the three‐tier system prediction and parallels with the most recent binary grading approaches. Furthermore, the OMRS model has the advantage of being a standardized procedure that performs with virtually no variability.

An growing body of evidence has demonstrated the success of AI, especially deep learning image analysis of tissue slides, in contributing to the diagnosis and prognosis of a range of diseases.^23^, ^33^, ^34^ However, despite advancements in AI technology during the last decade, there are few studies adopting deep learning techniques in the field of oral potentially malignant disorders. According to a recently published systematic review,^35^ only one study has evaluated the use of machine learning to predict cancer development risk in OL; Baik et al.^36^ developed their semi‐automated algorithm using Random Forests, which is a more traditional machine learning approach, but applied an algorithm training strategy similar to what we did in this study. Their algorithm learned from a set of normal oral mucosa and OC specimens how to differentiate normal from abnormal cell nuclei. Although their approach was highly effective, it relied on a special nucleus staining (Feulgen‐Thionin), which is not part of the routine H&E/surgical pathology‐staining protocols routine, and it was dependent on a skilled technician for identification and delineation of regions of interest in the tissue. Conversely, our algorithm was developed using an advanced deep learning technique, which autonomously identifies regions of interest from digital whole slide images and was trained to work with H&E‐stained slides using the standard staining protocol at surgical pathology services.

The OMRS model provides supporting evidence for the usefulness of deep learning algorithms using image feature extraction to contribute to the development of new diagnostic and prognostic tools that could potentially benefit patients with oral diseases. That said, we believe that by incorporating other data points into our algorithm, including clinical and genomic data, we may be able to build an even stronger OC risk prediction model, though a much larger dataset will be needed.

There are still limitations for future improvement in this study. The current training data set of nondysplastic oral epithelium and OSCC tissue images used for OMRS model development is small, which could lower the robustness of the currently trained OMRS model. More tissue sections of nondysplastic and dysplastic oral epithelium and OSCC, preferably from multiple centers should be incorporated into future models to improve performance. In addition, the prognostic study was only done in OL patients without concurrent oral cancer. It would be prudent to expand the study into other subsets of OPMD, and explore patients with or without concurrent oral cancer when a larger data set is available.

In summary, our study presents a new predictive tool that performs at least as well as the available OL histologic dysplasia grading approach, but with the additional advantage of being automated and free of variability. We believe that with the improvement of this model, it could potentially be an important tool for the early diagnosis of OC and safeguard those patients with a lower risk of malignant progression from unnecessary mental stress and recurring surgical intervention.

## AUTHOR CONTRIBUTIONS

**Xinyi Zhang:** Conceptualization (equal); data curation (equal); formal analysis (equal); investigation (equal); methodology (equal); validation (equal); visualization (equal); writing – original draft (equal); writing – review and editing (equal). **Frederico O. Gleber‐Netto:** Conceptualization (equal); data curation (equal); formal analysis (equal); investigation (equal); validation (equal); visualization (equal); writing – original draft (equal); writing – review and editing (equal). **Shidan Wang:** Conceptualization (equal); methodology (equal); supervision (equal). **Roberta Rayra Martins‐Chaves:** Data curation (equal); resources (equal); supervision (equal). **Ricardo Santiago Gomez:** Data curation (equal); resources (equal); writing – review and editing (equal). **Nadarajah Vigneswaran:** Data curation (equal); resources (equal); writing – review and editing (equal). **Arunangshu Sarkar:** Data curation (equal); writing – review and editing (equal). **William N. William:** Data curation (equal); resources (equal); writing – review and editing (equal). **Vassiliki A Papadimitrakopoulou:** Data curation (equal); resources (equal); writing – review and editing (equal). **Michelle D. Williams:** Data curation (equal); resources (equal); writing – review and editing (equal). **Diana Bell:** Data curation (equal); resources (equal); writing – review and editing (equal). **Doreen Palsgrove:** Data curation (equal); resources (equal); writing – review and editing (equal). **Justin Bishop:** Data curation (equal); supervision (equal); writing – review and editing (equal). **John Heymach:** Funding acquisition (equal); resources (equal); supervision (equal); writing – review and editing (equal). **Ann M. Gillenwater:** Data curation (equal); resources (equal); writing – review and editing (equal). **Jeffrey Myers:** Conceptualization (equal); funding acquisition (equal); supervision (equal); writing – review and editing (equal). **Renata Ferrarotto:** Data curation (equal); resources (equal); writing – review and editing (equal). **Scott Lippman:** Resources (equal); supervision (equal); writing – original draft (equal); writing – review and editing (equal). **Curtis Pickering:** Conceptualization (equal); data curation (equal); formal analysis (equal); funding acquisition (equal); investigation (equal); methodology (equal); project administration (equal); resources (equal); supervision (equal); validation (equal); visualization (equal); writing – original draft (equal); writing – review and editing (equal). **Guanghua Xiao:** Conceptualization (equal); formal analysis (equal); funding acquisition (equal); investigation (equal); methodology (equal); resources (equal); supervision (equal); validation (equal); visualization (equal); writing – original draft (equal); writing – review and editing (equal).

## FUNDING INFORMATION

This work has been supported by the National Institutes of Health (1R01GM140012, 1R01GM141519, 1R01DE030656, 1U01CA249245, and 2P30CA142543) and the Cancer Prevention and Research Institute of Texas (RP190107). The funding bodies had no role in the design, collection, analysis, or interpretation of data in this study.

## CONFLICT OF INTEREST STATEMENT

Vassiliki Papadimitrakopoulou is an employee and a shareholder of Pfizer, Inc. Other authors declare no conflict of interest.

## Supporting information


Appendix S1.

## Data Availability

Pathology images that support the findings of this study were available online in The Cancer Genome Atlas (TCGA, https://wiki.cancerimagingarchive.net/display/Public/).

## References

[1] Medical Intelligence. Edinb Med Surg J. 1806;2(7):376‐392. PMC5761374 30329989

[2] Speight PM , Khurram SA , Kujan O . Oral potentially malignant disorders: risk of progression to malignancy. Oral Surg Oral Med Oral Pathol Oral Radiol. 2018;125(6):612‐627. doi:10.1016/j.oooo.2017.12.011 29396319

[3] Aguirre‐Urizar JM . Lafuente‐Ibáñez de Mendoza I, Warnakulasuriya S. malignant transformation of oral leukoplakia: systematic review and meta‐analysis of the last 5 years. Oral Dis. 2021;27(8):1881‐1895. doi:10.1111/odi.13810 33606345

[4] Siegel RL , Miller KD , Jemal A . Cancer statistics, 2020. CA Cancer J Clin. 2020;70(1):7‐30. doi:10.3322/caac.21590 31912902

[5] Gerstner AO . Early detection in head and neck cancer ‐ current state and future perspectives. GMS Curr Top Otorhinolaryngol Head Neck Surg. 2008;7:Doc06. 22073093 PMC3199835

[6] Le Campion A , Ribeiro CMB , Luiz RR , et al. Low survival rates of Oral and oropharyngeal squamous cell carcinoma. Int J Dent. 2017;2017:5815493. doi:10.1155/2017/5815493 28638410 PMC5468590

[7] Warnakulasuriya S , Kujan O , Aguirre‐Urizar JM , et al. Oral potentially malignant disorders: a consensus report from an international seminar on nomenclature and classification, convened by the WHO collaborating Centre for Oral Cancer. Oral Dis. 2020;27:1862‐1880. doi:10.1111/odi.13704 33128420

[8] Reibel J . Prognosis of oral pre‐malignant lesions: significance of clinical, histopathological, and molecular biological characteristics. Crit Rev Oral Biol Med. 2003;14(1):47‐62. doi:10.1177/154411130301400105 12764019

[9] Lumerman H , Freedman P , Kerpel S . Oral epithelial dysplasia and the development of invasive squamous cell carcinoma. Oral Surg Oral Med Oral Pathol Oral Radiol Endod. 1995;79(3):321‐329. doi:10.1016/s1079-2104(05)80226-4 7621010

[10] Yan F , Reddy PD , Nguyen SA , Chi AC , Neville BW , Day TA . Grading systems of oral cavity pre‐malignancy: a systematic review and meta‐analysis. Eur Arch Otorhinolaryngol. 2020;277(11):2967‐2976. doi:10.1007/s00405-020-06036-1 32447493

[11] Kujan O , Oliver RJ , Khattab A , Roberts SA , Thakker N , Sloan P . Evaluation of a new binary system of grading oral epithelial dysplasia for prediction of malignant transformation. Oral Oncol. 2006;42(10):987‐993. 16731030 10.1016/j.oraloncology.2005.12.014

[12] Nankivell P , Williams H , Matthews P , et al. The binary oral dysplasia grading system: validity testing and suggested improvement. Oral Surg Oral Med Oral Pathol Oral Radio. 2013;115(1):87‐94. 10.1016/j.oooo.2012.10.015 23217539

[13] Abbey LM , Kaugars GE , Gunsolley JC , et al. Intraexaminer and interexaminer reliability in the diagnosis of oral epithelial dysplasia. Oral Surg Oral Med Oral Pathol Oral Radiol Endod. 1995;80(2):188‐191. doi:10.1016/s1079-2104(05)80201-x 7552884

[14] Bera K , Schalper KA , Rimm DL , Velcheti V , Madabhushi A . Artificial intelligence in digital pathology ‐ new tools for diagnosis and precision oncology. Nat Rev Clin Oncol. 2019;16(11):703‐715. doi:10.1038/s41571-019-0252-y 31399699 PMC6880861

[15] Wang S , Yang DM , Rong R , Zhan X , Xiao G . Pathology image analysis using segmentation deep learning algorithms. Am J Pathol. 2019;189(9):1686‐1698. doi:10.1016/j.ajpath.2019.05.007 31199919 PMC6723214

[16] LeCun Y , Bengio Y , Hinton G . Deep learning. Nature. 2015;521(7553):436‐444. doi:10.1038/nature14539 26017442

[17] Esteva A , Kuprel B , Novoa RA , et al. Dermatologist‐level classification of skin cancer with deep neural networks. Nature. 2017;542(7639):115‐118. doi:10.1038/nature21056 28117445 PMC8382232

[18] Xu J , Luo X , Wang G , Gilmore H , Madabhushi A . A deep convolutional neural network for segmenting and classifying epithelial and stromal regions in histopathological images. Neurocomputing. 2016;191:214‐223. doi:10.1016/j.neucom.2016.01.034 28154470 PMC5283391

[19] Sharma H , Zerbe N , Klempert I , Hellwich O , Hufnagl P . Deep convolutional neural networks for automatic classification of gastric carcinoma using whole slide images in digital histopathology. Comput Med Imaging Graph. 2017;61:2‐13. doi:10.1016/j.compmedimag.2017.06.001 28676295

[20] Coudray N , Ocampo PS , Sakellaropoulos T , et al. Classification and mutation prediction from non‐small cell lung cancer histopathology images using deep learning. Nat Med. 2018;24(10):1559‐1567. doi:10.1038/s41591-018-0177-5 30224757 PMC9847512

[21] Araújo T , Aresta G , Castro E , et al. Classification of breast cancer histology images using convolutional neural networks. PLOS One. 2017;12(6):e0177544. doi:10.1371/journal.pone.0177544 28570557 PMC5453426

[22] Ehteshami Bejnordi B , Veta M , Johannes van Diest P , et al. Diagnostic assessment of deep learning algorithms for detection of lymph node metastases in women with breast cancer. Jama. 2017;318(22):2199‐2210. doi:10.1001/jama.2017.14585 29234806 PMC5820737

[23] Steiner DF , MacDonald R , Liu Y , et al. Impact of deep learning assistance on the histopathologic review of lymph nodes for metastatic breast cancer. Am J Surg Pathol. 2018;42(12):1636‐1646. doi:10.1097/PAS.0000000000001151 30312179 PMC6257102

[24] Liu Y , Gadepalli K , Norouzi M , et al. Detecting cancer metastases on gigapixel pathology images. arXiv Preprint arXiv. 2017;170302442.

[25] Krizhevsky A , Sutskever I , Hinton GE . Imagenet classification with deep convolutional neural networks. Adv Neural Inf Process Syst. 2012;25:1097‐1105.

[26] Albawi S , Mohammed TA , Al‐Zawi S , eds. Understanding of a Convolutional Neural Network. 2017 International Conference on Engineering and Technology (ICET). IEEE; 2017:21‐23.

[27] Litjens G , Kooi T , Bejnordi BE , et al. A survey on deep learning in medical image analysis. Med Image Anal. 2017;42:60‐88. doi:10.1016/j.media.2017.07.005 28778026

[28] Zuley ML , Jarosz R , Kirk S , et al. Radiology data from the cancer genome atlas head‐neck squamous cell carcinoma [TCGA‐HNSC] collection. Cancer Imaging Arch. 2016;10:K9.

[29] Szegedy C , Vanhoucke V , Ioffe S , Shlens J , Wojna Z , eds. Rethinking the inception architecture for computer vision. Proceedings of the IEEE Conference on Computer Vision and Pattern Recognition. IEEE; 2016.

[30] Vigneswaran N , Muller S , Williams M , Lippman S . Benign Leukoplakia‐like Lesions. In: El‐Naggar A , Grandis J , Slootweg P , Chan J , Takata T , eds. World Health Organization Classification of Tumours. 4th ed. IARC Press; 2017.

[31] Dong Y , Chen Y , Tao Y , et al. Malignant transformation of oral leukoplakia treated with carbon dioxide laser: a meta‐analysis. Lasers Med Sci. 2019;34(1):209‐221. 30443884 10.1007/s10103-018-2674-7

[32] Mehanna HM , Rattay T , Smith J , McConkey CC . Treatment and follow‐up of oral dysplasia—a systematic review and meta‐analysis. Head Neck. 2009;31(12):1600‐1609. 19455705 10.1002/hed.21131

[33] Wang S , Rong R , Yang DM , et al. Computational staining of pathology images to study the tumor microenvironment in lung cancer. Cancer Res. 2020;80(10):2056‐2066. doi:10.1158/0008-5472.Can-19-1629 31915129 PMC7919065

[34] Kiani A , Uyumazturk B , Rajpurkar P , et al. Impact of a deep learning assistant on the histopathologic classification of liver cancer. NPJ Digit Med. 2020;3(1):23. doi:10.1038/s41746-020-0232-8 32140566 PMC7044422

[35] Mahmood H , Shaban M , Indave BI , Santos‐Silva AR , Rajpoot N , Khurram SA . Use of artificial intelligence in diagnosis of head and neck precancerous and cancerous lesions: a systematic review. Oral Oncol. 2020;110:104885. doi:10.1016/j.oraloncology.2020.104885 32674040

[36] Baik J , Ye Q , Zhang L , et al. Automated classification of oral premalignant lesions using image cytometry and random forests‐based algorithms. Cell Oncol. 2014;37(3):193‐202. 10.1007/s13402-014-0172-x PMC13004423 24817187

