# Supplementary material for: Deep learning‐based pathology image analysis predicts cancer progression risk in patients with oral leukoplakia
Source: Cancer Med. 2023 Jan 31;12(6):7508–18. doi: 10.1002/cam4.5478 (PMC10067069; doi:10.1002/cam4.5478)
Supplement: Supplementary file 1 — Appendix S1. [file CAM4-12-7508-s001.docx]

**Supplemental methods**

***ORMS model establishment - training process***

The OMRS model adapted a modified Inception (V3)^1^ architecture with input size 300×300 and weights pre-trained on ImageNet^2^ (including 3.2 million 3-channel images for classifying real-world images into different classes) and fine-tuned using our training set. The model took as input an image patch and output a patch-level probability of the 4 classes (low-risk epithelium, high-risk epithelium, connective tissue and background). Layers of flatten, dense, dropout (dropout rate = 0.7, only in training stage) and another dense layer were added to the end of the model for the prediction needed in our study. The network was trained with stochastic gradient descent algorithms in Keras with TensorFlow backend. The batch size was set to 64, the learning rate was set to 0.0001 without decay, and the momentum was set to 0.9.

H&E staining can vary between different processing batches, and some color variation can occur causing significant decrease in model performance. To minimize the negative effect of inconsistent staining on the training results, images were augmented intensely before being run through the model. Each image has a 70% chance to be augmented. Augmentation include Gaussian blur, random projective transformations, color shifting for the whole image, color shifting and variation for each color channel, and random flipping horizontally and vertically. The maximum number of epochs (the number of passes of the entire training dataset) to train was set to 100. To avoid overfitting, the training process was set to automatically stop after validation accuracy failed to improve for 10 epochs. The weights after the last training epoch were saved to be used for further analysis.

**Supplementary Table 1.** Diagnosis of oral mucosa H&E slides with normal oral epithelium.

| **Sample** | **Group** | **Diagnosis** | **Epithelial status** | **Connective tissue status** |
| --- | --- | --- | --- | --- |
| 1 | Connective tissue disease - not cancer related | Fibrous hyperplasia | Normal | Altered |
| 2 |  | Fibrous hyperplasia | Normal | Altered |
| 3 |  | Fibrous hyperplasia | Normal | Altered |
| 4 |  | Intramuscular lipoma | Normal | Altered |
| 5 |  | Oral mucosa and Mucocele | Normal | Altered |
| 6 |  | Peripheral ossifying fibroma | Normal | Altered |
| 7 | Normal mucosa | Normal mucosa | Normal | Normal |
| 8 |  | Normal mucosa and sialoadenitis | Normal | Altered |
| 9 |  | Normal mucosa fragment | Normal | Normal |
| 10 |  | Normal mucosa with focal inflammatory infiltrate | Normal | Altered |
| 11 |  | Normal mucosa with inflammatory infiltrate | Normal | Altered |
| 12 |  | Oral mucosa and Ranula | Normal | Normal |
| 13 |  | Oral mucosa with nonspecific chronic inflammatory process | Normal | Altered |

**Supplementary Table 2.** Patient characteristics of 25 oral mucosa H&E slides with cancerous epithelium from TCGA.

| (*n* = 25) | | |
| --- | --- | --- |
| Sex, *n* (%) |  |  |
| Male | 18 | 72.0% |
| Female | 7 | 28.0% |
| Age (days) |  |  |
| Median, *n* (range) | 24778 | 7301-31242 |
| Race, *n* (%) |  |  |
| Black or African American | 2 | 8.0% |
| White | 20 | 80.0% |
| Asian | 2 | 8.0% |
| Unknown or not reported | 1 | 4.0% |
| Primary tumor |  |  |
| T2 | 5 | 20.0% |
| T3 | 6 | 24.0% |
| T4 | 13 | 52.0% |
| TX | 1 | 4.0% |
| Lymph node |  |  |
| Positive (%) | 12 | 48.0% |
| Negative (%) | 12 | 48.0% |
| NA | 1 | 4.0% |
| Distant metastasis |  |  |
| Positive (%) | 0 | 0.0% |
| Negative (%) | 24 | 96.0% |
| NA | 1 | 4.0% |
| Clinical stage |  |  |
| Stage II | 4 | 16.0% |
| Stage III | 3 | 12.0% |
| Stage IV | 17 | 68.0% |
| NA | 1 | 4.0% |


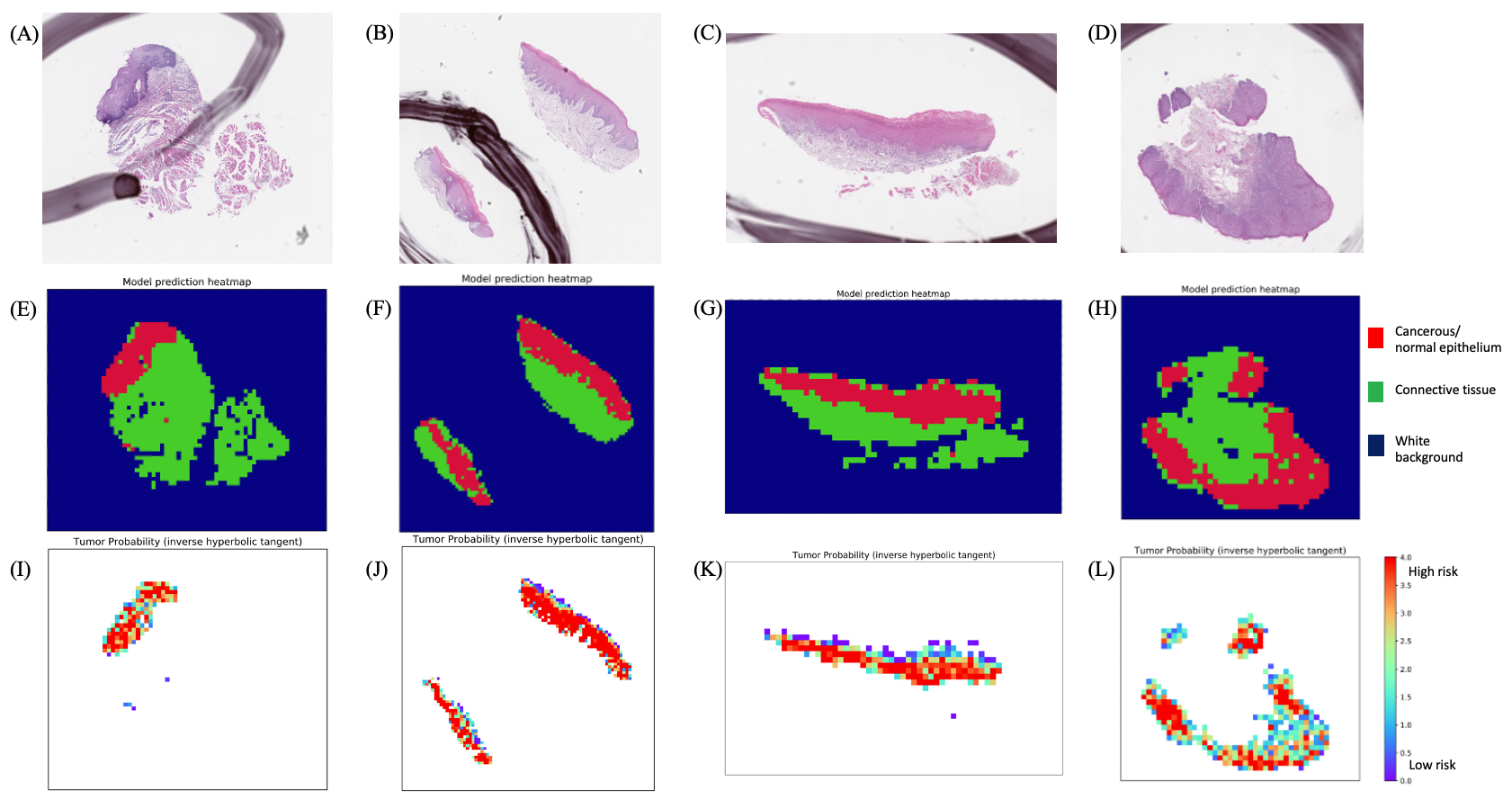


**Supplementary Figure 1.** More example results of image-level region detection results on oral leukoplakia pathology images.

**(A)-(D)** Original image. **(E)-(H)** Predicted region labels. Red: tumor or non-dysplastic epithelium; green: connective tissue; blue: white background region. **(I)-(L)** Predicted risk probability.

Each point in the region label maps and cancerous epithelium probability heatmaps corresponds to a 300×300 pixel image patch in original 40x image.

1. Szegedy C, Vanhoucke V, Ioffe S, Shlens J, Wojna Z. Rethinking the inception architecture for computer vision. 2016:2818-2826.

2. Deng J, Dong W, Socher R, Li L-J, Li K, Fei-Fei L. Imagenet: A large-scale hierarchical image database. Ieee; 2009:248-255.
